# The physiological impact of an N‐terminal Halo‐tag on glucose‐dependent insulinotropic polypeptide receptor function in mice

**DOI:** 10.1111/dom.16216

**Published:** 2025-01-30

**Authors:** Iona Davies, Yusman Manchanda, Kyle W. Sloop, Stephen R. Bloom, Tricia M.‐M. Tan, Alejandra Tomas, Ben Jones

**Affiliations:** ^1^ Section of Endocrinology and Investigative Medicine, Department of Metabolism, Digestion and Reproduction, Faculty of Medicine Imperial College London London UK; ^2^ Section of Cell Biology and Functional Genomics, Department of Metabolism, Digestion and Reproduction, Faculty of Medicine Imperial College London London UK; ^3^ Diabetes, Obesity and Complications, Lilly Research Laboratories Eli Lilly and Company Indianapolis Indiana USA

**Keywords:** GIP, incretin physiology, mouse model, type 2 diabetes

## 1 INTRODUCTION

The role of glucose dependent insulinotropic polypeptide (GIP) signalling in metabolic homeostasis is a hot area of research. The study of GIP receptor (GIPR) expression and trafficking, vital aspects in understanding GIPR physiology and pharmacology, has been hindered by the lack of validated GIPR antibodies, meaning that studies have relied on the presence of *Gipr* mRNA to determine GIPR expression in mouse and human tissue.^1^, ^2^, ^3^ The development of fluorophore‐conjugated GIPR agonists has provided some insights into endogenous GIPR distribution and behaviour^3^, ^4^ but offers only indirect evidence of receptor localisation and may be misleading in some contexts, for example, if agonists dissociate from receptors after entering the endocytic pathway.

G protein‐coupled receptors (GPCRs) modified with self‐labelling enzymatic tags such as SNAP, Halo and CLIP are becoming increasingly common in pharmacological research to study receptor expression and trafficking in vitro.^5^, ^6^, ^7^ Advances in genetic engineering have enabled the generation of mice which endogenously express proteins tagged with enzyme self‐labels, such as the *Glp1r* ^*SNAP/SNAP*^ mouse model.^8^ This model was used to show that pancreatic islet GLP‐1R constitutive internalisation was higher than reported using cell lines, emphasising the importance of studying receptor behaviours in their native environment.

To this end, we developed a *Gipr* ^*Halo/Halo*^ mouse model for the study of endogenous GIPR expression and trafficking.

## 2 METHODS

Methodology is provided in the Supplementary data.

## 3 RESULTS

**FIGURE 1:**
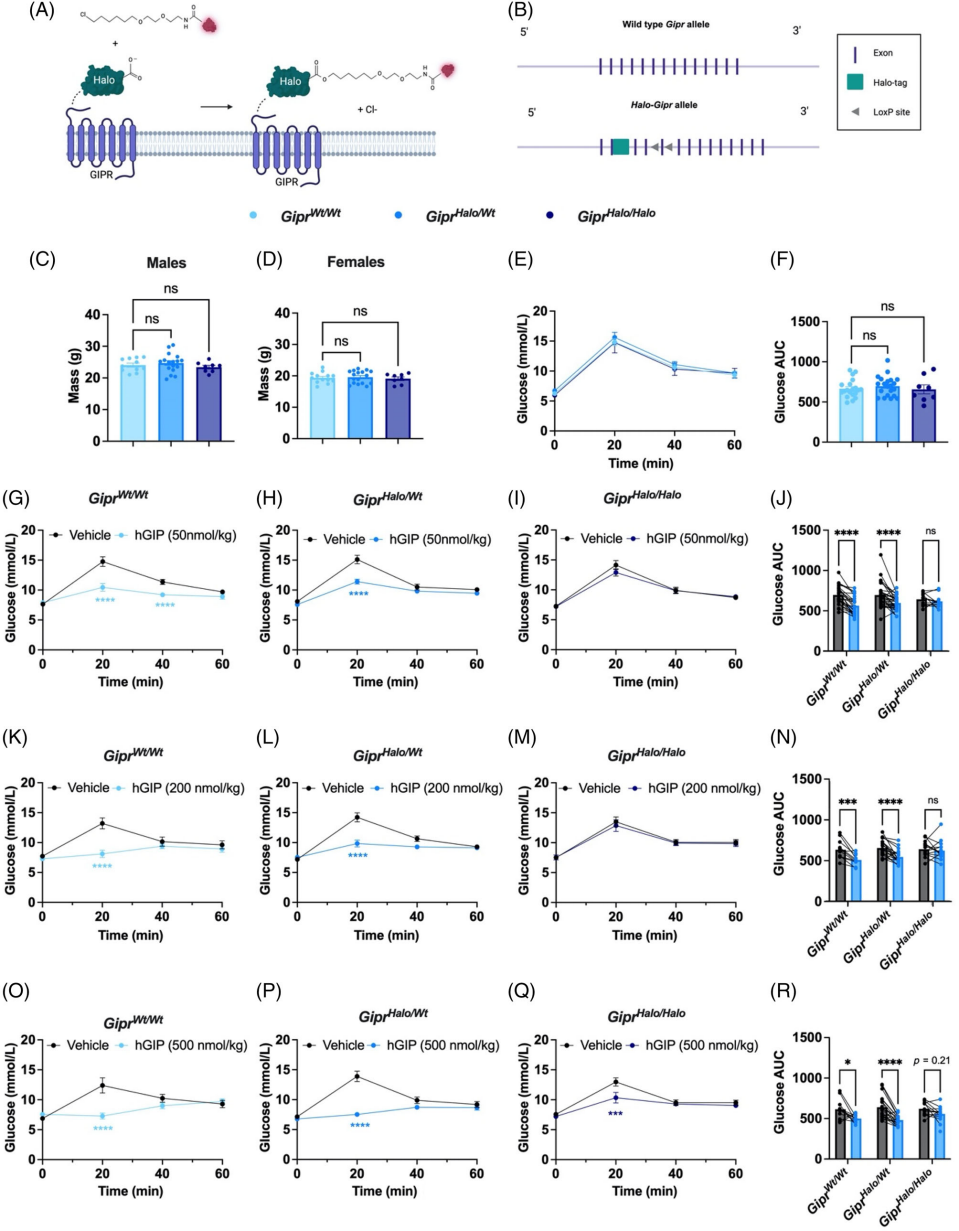
N‐terminally Halo‐tagged glucose dependent insulinotropic polypeptide receptor (GIPR) displays a partially impaired receptor function in vivo. (A) Schematic of GIPR tagging with Halo‐tag protein and subsequent receptor labelling. (B) Wild‐type *Gipr* and *Halo‐Gipr* alleles. (C, D) Body mass (g) of *Gipr* ^*Wt/Wt*^ (male: *N* = 11, female: *N* = 13), *Gipr* ^*Halo/Wt*^ (male: *N* = 17, female: N = 17) and *Gipr* ^*Halo/Halo*^ (male: *N* = 8, female: *N* = 8), measured at 6 weeks of age. Males are displayed in (C) and females in (D). (E, F) Oral glucose tolerance test (OGTT) conducted in *Gipr* ^*Wt/Wt*^ (*n* = 20), *Gipr* ^*Halo/Wt*^ (*n* = 21) and *Gipr* ^*Halo/Halo*^ (n = 8) mice. (G–R) Crossover Intraperitoneal glucose tolerance tests (IPGTTs) conducted in *Gipr* ^*Wt/Wt*^ mice (G: *N* = 24, K: *N* = 11, O: *N* = 11), *Gipr* ^*Halo/Wt*^ mice (H: *N* = 33, L: *N* = 20, P: *N* = 20) and *Gipr* ^*Halo/Halo*^ mice (I: *N* = 12, M: *N* = 14, Q: *N* = 13–14), in response to human GIP (hGIP, 50 nmol/kg) (G–J), hGIP (200 nmol/kg) (K–N) and hGIP 500 nmol/kg (O–R). (E, G–I, K–M, O–Q) Plasma glucose time‐course. (F, J, N, R) Glucose area under the curve (AUC) derived from corresponding glucose curves. Blood glucose at specific time‐points were analysed using a two‐way analysis of variance (ANOVA) with time and subgroup as co‐variables. Šídák test used to correct for multiple comparisons. Body mass at 6 weeks (C, D) and glucose AUC in (F) were analysed with a one‐way ANOVA. Dunnett's test was used to correct for multiple comparisons. Glucose AUCs in (J), (N) and (R) were analysed using a two‐way ANOVA with genotype and subgroup as co‐variables. Šídák test was used to correct for multiple comparisons. All values are presented as a mean ± SEM. *p < 0.05, ***p < 0.001, ****p < 0.0001.

N‐terminal tags facilitate cell surface‐specific labelling (Figure 1A). Previous studies using heterologous expression systems show that GIPR tolerates N‐terminal Halo‐ and SNAP‐tag modifications.^9^, ^10^ To adapt this approach to natively expressed GIPR, we designed a *Gipr* ^*Halo/Halo*^ mouse model, in which the sequence for the Halo‐tag protein was inserted into the *Gipr* allele downstream of the GIPR signal peptide in exon 2, and *loxP* sites inserted between exon 5 (Figure 1B) via ES‐cell‐based gene targeting.

To establish whether Halo‐tagging affects physiological GIPR signalling, we investigated differences in body weight gain and handling of oral glucose administration between chow‐fed Halo‐tagged and wild‐type genotypes. There were no significant differences in body weight between genotypes of either sex at 6 weeks (Figure 1C,D), or between 6 and 9 weeks measured in a subset of mice (Figure S1). This does not preclude central GIPR signalling disturbances as body weight differences between *Gipr* KO and wild‐type mice are only revealed upon high‐fat diet feeding.^11^ Plasma glucose responses to an oral glucose bolus were identical across all genotypes (Figure 1E,F). However, *Gipr* ^*Halo/Halo*^ mice showed impaired sensitivity to human GIP (hGIP) administered exogenously during an intraperitoneal glucose tolerance test, requiring a 10 times higher dose to improve glucose tolerance than was needed for *Gipr* ^*Wt/Wt*^ and *Gipr* ^*Halo/Wt*^ mice, suggestive of impaired pancreatic GIPR functionality (Figure 1G–R). The same trends were present across both sexes but did not reach statistical significance in all cases (Figures S2 and S3).

**FIGURE 2:**
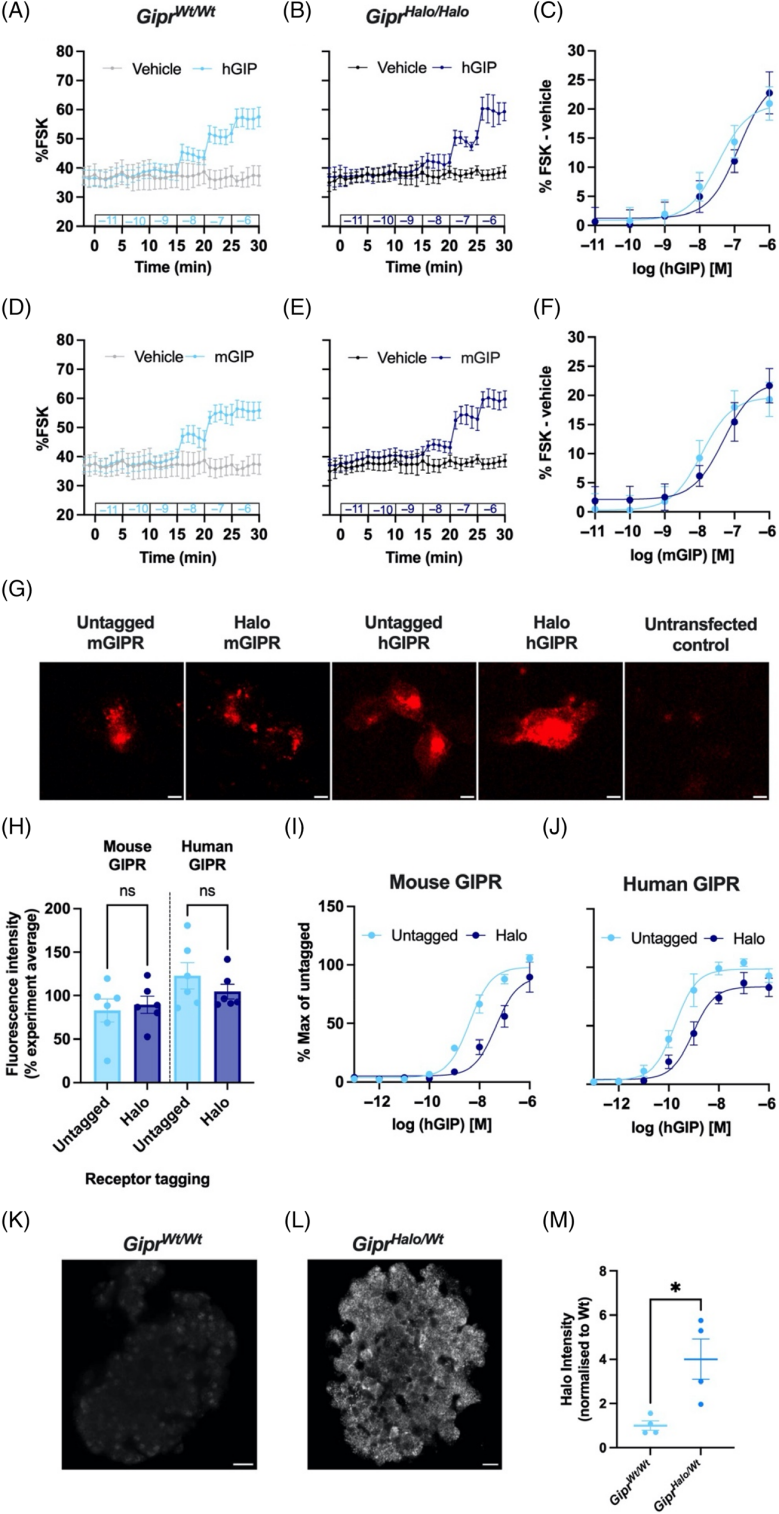
Investigating the causes of reduced functionality of the Halo‐tagged glucose dependent insulinotropic polypeptide receptor (GIPR) and visualising endogenous GIPR via anti‐Halo antibody staining. (A–F) cAMP signal as a percentage of FSK/IBMX responses in cADDis‐transduced dispersed islet cells from *Gipr* ^*Wt/Wt*^ mice (*n* = 3–4) (A, D) or *Gipr* ^*Halo/Halo*^ mice (*n* = 4) (B, E), in response to stepwise addition of human GIP (hGIP; A, B) and mouse GIP (mGIP; D, E). (C, F) cAMP dose–response curves derived from area under the curve in each time interval in (A) and (B) (for C) and (D) and (E) (for F) minus vehicle control, with three parameter fits shown. (G) Representative images of experiment displayed in (H) (scale bars = 20 μm). (H) Fluorescence intensity of AD293 cells transiently transfected with mouse and human untagged and Halo‐tagged GIPR following 1 h of labelling with GIP‐TMR (100 nM) (*n* = 6), expressed as a percentage of the experimental average. Data were analysed using a one‐way analysis of variance. The Šídák test was used to correct for multiple comparisons. (I, J) cAMP dose responses in AD293 cells transiently transfected with mouse (I) and human (J) untagged and Halo‐tagged GIPR, stimulated for 30 min with increasing doses of hGIP (*n* = 4–5). Three parameter fits are shown, with values normalised to the % max of the mouse or human untagged receptor. (K, L) Representative images of a pancreatic islet from *Gipr* ^*Wt/Wt*^ (K) and *Gipr* ^*Halo/Wt*^ (L) mice labelled with an anti‐Halo antibody. Scale bar = 20 μm. (M) Mean intensity of cell surface anti‐Halo staining normalised to wild‐type from 9 to 21 islets from *Gipr* ^*Wt/Wt*^ (*n* = 4) versus littermate *Gipr* ^*Halo/Wt*^ (*n* = 4) mice. Changes in fluorescence intensity were analysed using Student's *t* test. Values are presented as a mean ± SEM; *p < 0.05. hGIP, human GIP; mGIP, mouse GIP.

In view of the reduced anti‐hyperglycaemic response to hGIP in *Gipr* ^*Halo/Halo*^ mice, we analysed cAMP signalling dynamics in dispersed pancreatic islets from *Gipr* ^*Wt/Wt*^ and *Gipr* ^*Halo/Halo*^ mice transduced with the fluorescence‐based cADDis cAMP biosensor and stimulated with a stepwise concentration gradient of hGIP or mouse GIP (mGIP) (Figure 2A–F). In accordance with our in vivo glucose tolerance results, islets from *Gipr* ^*Halo/Halo*^ mice still responded to GIP, but there was a statistically significant reduction in potency compared to islets from *Gipr* ^*Wt/Wt*^ mice (mGIP EC_50_: *Gipr* ^*Wt/Wt*^ = 11.4 nM, *Gipr* ^*Halo/Halo*^ = 72.2 nM [*p* = 0.01 by unpaired *t* test of logEC_50_s]; hGIP EC_50_: *Gipr* ^*Wt/Wt*^ = 42.4 nM, *Gipr* ^*Halo/Halo*^ = 146.8 nM [*p* = 0.07 by unpaired *t* test of logEC_50_s]).

We wondered if this impairment in cAMP signalling could be specific to endogenously expressed Halo‐GIPR, which might be expected to reveal subtle differences in function unseen in heterologous systems. Therefore, we performed cAMP assays using AD293 cells transiently transfected with either untagged or Halo‐tagged human or mouse GIPR. High‐content cell surface labelling with the fluorescent GIP analogue GIP‐TMR^10^ suggested no impact of the Halo‐tag on delivery of the human or mouse GIPR to the plasma membrane (Figure 2G,H). However, in keeping with our ex vivo results from dispersed mouse islets, cells expressing the Halo‐mGIPR displayed an impaired cAMP response to hGIP compared with untagged mGIPR (untagged mGIPR EC_50_ = 6.0 nM, Halo‐mGIPR = 55.5 nM, *p* = 0.01 by paired *t* test of logEC_50_s), a finding also observed at the human GIPR albeit to a lesser extent (untagged hGIPR EC_50_ = 0.4 nM, Halo‐hGIPR = 1.6 nM, *p* = 0.03 by paired *t* test of logEC_50_s) (Figure 2I,J). Thus, these results suggest that in in vitro models, N‐terminal Halo‐tagging of the GIPR may have either affected ligand binding or intracellular G protein signalling from the receptor.

As exogenously expressed Halo‐GIPR was expressed and trafficked to the cell surface in heterologous cells, we tested whether our Halo‐GIPR mouse model could be used to visualise pancreatic islet GIPR expression by Halo‐tag detection, a tissue chosen due to relatively high levels of *Gipr* expression. Unfortunately, initial attempts to label islets using Halo‐probes were unsuccessful. While the use of improved Halo‐probes may be a possibility for the future,^9^ we employed an antibody‐based immunofluorescence approach which would potentially afford increased sensitivity from signal amplification; indeed, anti‐Halo antibody staining revealed a significantly higher intensity of signal at the surface of islet cells in *Gipr* ^*Halo/Wt*^ islets compared to *Gipr* ^*Wt/Wt*^ islets (Figure 2K–N). Although endogenous islet GIPR expression has previously been inferred via fluorophore‐conjugated agonists,^10^ this is the first evidence of direct detection of endogenous GIPR in pancreatic islets using antibody labelling.

Interestingly, we were unable to detect a signal in *Gipr* ^*Halo/Halo*^ islets (Figure S4), suggesting that, unlike in overexpressing in vitro systems (Figure 2G,H), endogenously expressing both *Halo‐Gipr* alleles appears to sufficiently alter receptor biosynthetic trafficking to prevent cell surface Halo‐GIPR detection. This offers a potential explanation of our in vivo and ex vivo observation of impaired Halo‐GIPR function in response to GIP stimulation.

## 4 DISCUSSION

We generated a *Gipr* ^*Halo/Halo*^ mouse model for the study of endogenous GIPR expression and trafficking. However, in and ex vivo analysis of pancreatic responses to pharmacological levels of GIP revealed that the N‐terminally Halo‐tagged GIPR displayed impaired function. Impaired signalling in heterologous cells transfected with the Halo‐tagged GIPR despite normal cell surface expression, combined with an inability to detect cell surface Halo‐expression in *Gipr* ^*Halo/Halo*^ islets, suggests that impaired Halo‐GIPR responses *in and* ex vivo could be due to both reduced cell surface receptor expression and impaired downstream signal transduction. In view of clear loss of GIP sensitivity, we suspect that normal glucose responses in *Gipr* ^*Halo/Halo*^ mice during an oral glucose tolerance test reflects upregulation of GLP‐1R signalling to compensate for the germline impairment of GIPR function.^12^

In conclusion, our results serve as a cautionary tale to the use of enzyme self‐labels in endogenous GPCR labelling. While we advise against the use of *Gipr* ^*Halo/Halo*^ mice to study GIPR functional responses, our success in labelling endogenous GIPR in pancreatic islets suggests that this model could still be a valuable tool in evaluating GIPR expression in tissues where this is contentious, such as adipose tissue and the CNS.^1^, ^2^, ^3^, ^4^

## CONFLICT OF INTEREST STATEMENT

KWS is an employee of Eli Lilly and Company and may own company stock. SRB is an employee of Zihipp Ltd. TM‐MT is a former consultant for and shareholder in Zihipp Ltd. AT has received funding from Eli Lilly and Company and Sun Pharma. BJ has received funding from Eli Lilly and Company, Metsera Inc and Sun Pharma, and acts as a consultant for Metsera Inc.

### PEER REVIEW

The peer review history for this article is available at https://www.webofscience.com/api/gateway/wos/peer‐review/10.1111/dom.16216.

## Supporting information


**Data S1.** Supporting information.

## Data Availability

The data that support the findings of this study are available from the corresponding author upon reasonable request.
