# Supplementary material for: The physiological impact of an N‐terminal Halo‐tag on glucose‐dependent insulinotropic polypeptide receptor function in mice
Source: Diabetes Obes Metab. 2025 Jan 30;27(4):2294–8. doi: 10.1111/dom.16216 (PMC11885072; doi:10.1111/dom.16216)
Supplement: Supplementary file 1 — Data S1. Supporting information. [file DOM-27-2294-s001.docx]

**Supplementary Methods**

**Peptides**

Mouse GIP(1-42) and human GIP(1-42) were purchased from Bachem, Switzerland. GIP-TMR, a previously described fluorescent analogue of human GIP(1-42) (1), was purchased from Wuxi AppTec, China.

**Cell culture**

AD293 cells (Agilent, US) were maintained in Dulbecco’s modified medium (DMEM, Thermo Fisher, UK) with 1% penicillin/streptomycin (P/S, Sigma, UK) and 10% foetal bovine serum (FBS, Thermo Fisher, UK) (complete DMEM).

**Plasmids**

Plasmids encoding full length wild-type human and mouse GIPR in the pcDNA/FRT vector (Thermo Fisher, UK), and human and mouse GIPR featuring an N-terminal extracellular Halo-tag in the pcDNA5/FRT vector were custom synthesised by Genewiz, UK. The Halo-tag was placed downstream of the native human or mouse GIPR signal peptides.

**Homogenous Time Resolved Fluorescence (HTRF) cAMP accumulation assay**

HTRF cAMP accumulation assays were performed using the cAMP-Gs Dynamic 2 kit (Cisbio, France) as per the manufacturer’s protocol in AD293 cells 24 hours after transient transfection with specified plasmid. Full details have been described previously (2).

**Cell surface labelling**

24 hours post transient transfection with specified plasmid, AD293 cells were labelled for 1 hour with GIP-TMR (100 nM) in DMEM + 0.1% bovine serum albumin (BSA), prior to fixation with 1% paraformaldehyde and imaging using an epifluorescence microscope.

***In vivo* studies – husbandry**

All animal procedures were approved by the British Home Office UK Animals (Scientific Procedures) Act 1986. All experiments were performed using both female and male *Gipr^Wt/Wt^*, *Gipr^Halo/Wt^* and *Gipr^Halo/Halo^* 6–24 week-old littermates. For all *in vivo* studies, mice were group housed in individually ventilated cages with a standard 12:12 hour light-dark cycle. Unless fasted, mice had free access to food (RM1- Special Diet Services, UK) and water.

**Generation of *Gipr^Halo/Halo^* mice**

*Gipr^Halo/Halo^* mice generation was outsourced to Cyagen. ES cell-based gene targeting was employed. The Halo tag cassette was inserted downstream of the mouse GIPR signal peptide (aa.1-18). *loxP* sites were inserted either side of exon 5. A neomycin resistance cassette was chosen as the positive selection marker and diphtheria toxin A as a negative selection marker. The targeting construct was electroporated into C57BL/6N ES cells and southern blot analysis was carried out to identify PCR-positive clones. Clones were then micro-injected into host embryos and transferred into surrogate mothers. Following subsequent chimera breeding and identification of heterozygous pups, sperm samples from two 11-week-old male heterozygous mice were cryopreserved and subsequently transferred to ES Cell & Transgenic Facility of the Laboratory of Medical Sciences, Medical Research Council where the mouse line was rederived on a C57BL/6J background using *in vitro* fertilisation.

**Genotyping**

Genotyping was performed using the KAPA Mouse Genotyping Kit (Sigma, UK) as per the manufacturer’s instructions. To identify the presence of the Halo-tag, a primer set for the Halo construct (forward: GATCCCAGCCTCACTTATCTACTG, reverse: GACCAACATCGACGTAGTGCAT) (expected fragment of 364 base pairs) and a primer set for a constitutively expressed cDNA sequence (forward: GCAGAAGAGGACAGATACATTCAT, reverse: CCTACTGAAGAATCTATCCCACAG) (hexokinase, expected fragment of 689 base pairs) were used. To identify the presence of the *loxP* sites between exon 5 (and thus confirm whether a mouse is *Gipr^Halo/Wt^* or *Gipr^Halo/Halo^*), a primer set was used where the wild-type exon 5 allele has an expected fragment of 281 base pairs and the *loxP*-flanked exon 5 allele has an expected fragment of 337 base pairs (forward: TGGGCTCTGTCACATGATTTACTTA, reverse: GAGGGTGTCAGAGGTGTAGC).

**Intraperitoneal glucose tolerance tests (IPGTTs)**

Mice were fasted at 08:00 hours prior to intraperitoneal glucose injection ± human GIP at a specified dose at 14:00 hours. The injection volume was adjusted to the body weight of the mouse so that all mice received glucose at 2 g/kg. Blood glucose measurements were taken via tail venesection at t=0, t=20, t=40 and t=60 minutes. Glucose readings were measured in mmol/L using a GlucoRx glucometer.

All studies were conducted as crossover studies. Three litters of mice were used for these studies: cohort 1 (*Gipr^Wt/Wt^*, male n=10, female n=10; *Gipr^Halo/Wt^*, male n=13, female n=8; *Gipr^Halo/Halo^*, male n=4, female n=4), cohort 2 (*Gipr^Wt/Wt^*, male n=1, female n=3; *Gipr^Halo/Wt^*, male n=4, female n=8; *Gipr^Halo/Halo^*, male n=1, female n=3) and cohort 3 (*Gipr^Wt/Wt^*, male n=2, female n=1; *Gipr^Halo/Wt^*, male n=1, female n=2; *Gipr^Halo/Halo^*, male n=4, female n=2). For studies testing hGIP (50 nmol/kg), cohorts 1 and 2 were used. For studies testing hGIP (200 nmol/kg), some of cohort 1 and all of cohort 2 and 3 were used. For studies testing hGIP (500 nmol/kg), some of cohort 1 and all of cohort 2 and 3 were used. Of note, the study conducted with cohort 3 was a three-way crossover study administering vehicle, hGIP (200 nmol/kg) and hGIP (500 nmol/kg) with glucose injection. Thus, when data was combined to separately compare vehicle *versus* hGIP (200 nmol/kg) and vehicle *versus* hGIP (500 nmol/kg) across all cohorts, the data collected from the saline group of cohort 3 was used in both analyses.

**Oral glucose tolerance tests (OGTTs)**

Cohort 1 was used for OGTTs. Mice were fasted at 08:00 hours prior to oral gavage of glucose at 14:00 hours, with the injection volume adjusted to the body weight of the mouse so that all mice received glucose at 2 g/kg. Blood glucose measurements were taken via tail venesection at t=0, t=20, t=40 and t=60 minutes. Glucose readings were measured in mmol/L using a GlucoRx glucometer.

***Ex vivo* islet dose responses**

Pancreatic islets from *Gipr^Wt/Wt^* and *Gipr^Halo/Halo^* mice were isolated as previously described (2), dispersed into single cells via 3-minute trituration with warm 0.05% trypsin-EDTA, and resuspended in RPMI-1640 (Thermo Fisher, UK) + 10% FBS + 0.1% P/S. Cells were transduced with the Green Up cADDis biosensor (Molecular Montana, US) (3) prior to seeding on 96-well plates coated with 0.01% poly-D-lysine hydrobromide and 25 µg/mL mouse laminin. Following overnight incubation (37°C, 95%:5% O_2_ :CO_2_ ratio), cells were imaged in Krebs-Ringer Bicarbonate buffer (140 mM NaCl, 3.6 mM KCl, 1.5 mM CaCl_2_, 0.5 mM MgSO_4_, 0.5 mM NaH_2_PO_4_, 2 mM NaHCO_3_, 10 mM HEPES, saturated with 95% O_2_/5% CO_2_, pH 7.4) with 6 mM glucose and 0.1% BSA at 37°C using an automated epifluorescence microscope, enabling multiple fields of view to be captured in parallel. Acquisitions were 36 minutes in length, consisting of a 3-minute baseline recording pre-peptide addition, 6 x 5-minute recordings following addition of 10-fold increasing concentrations of peptide and a final 3-minute recording following addition of 3-isobutyl-1-methylxanthine (IBMX) (500 µM) and forskolin (FSK) (50 µM) to maximally stimulate the sensor. cADDis signal quantification was performed using Fiji v1.54f (NIH), where islet cell fluorescence was normalised to both baseline and IBMX/FSK responses, and the area under the curve (AUC) calculated for each cell.

***Ex vivo* islet immunohistochemistry**

Pancreatic islets from *Gipr^Wt/Wt^*, *Gipr^Halo/Wt^* and *Gipr^Halo/Halo^* mice were isolated. Islets were fixed in paraformaldehyde (4%) and blocked without permeabilization in 1% BSA prior to 48-hour incubation with anti-Halo antibody (1:500; Promega, UK) in 0.1% BSA/PBS at 4°C. Islets were then washed with 3xPBS and incubated with secondary anti-rabbit AlexaFluor 568 (1:500; Thermo Scientific, UK). Islets were imaged using a Leica Stellaris 8 inverted confocal microscope with a 63x/1.40 oil objective with Lightning super-resolution modality from the Facility for Imaging by Light Microscopy (FILM) at Imperial College London.

**Statistical analysis**

Analyses was conducted using Prism 10.0 (GraphPad software). Statistical significance was calculated using one sample t-test, one- or two-way ANOVA, as indicated in the figure legends. Šídák and Dunnett’s tests were used to correct for multiple comparisons. Unless specified otherwise, all summarised data points are presented as mean ± SEM. For concentration response experiments, 3-parameter fits are plotted. Statistical significance was determined as *P<0.05, **P<0.01, ***P<0.001 and ****P<0.0001.

**Method references**

1. Manchanda Y, Bitsi S, Chen S, Broichhagen J, Bernardino De La Serna J, Jones B, et al. Enhanced Endosomal Signaling and Desensitization of GLP-1R vs GIPR in Pancreatic Beta Cells. Endocrinology (United States). 2023 May 1;164(5).

2. Hinds CE, Peace E, Chen S, Davies I, El Eid L, Tomas A, et al. Abolishing β-arrestin recruitment is necessary for the full metabolic benefits of G protein-biased glucagon-like peptide-1 receptor agonists. Diabetes Obes Metab. 2024 Jan 1;26(1):65–77.

3. Tewson PH, Martinka S, Shaner NC, Hughes TE, Quinn AM. New DAG and cAMP Sensors Optimized for Live-Cell Assays in Automated Laboratories. J Biomol Screen. 2016 Mar 1;21(3):298–305.


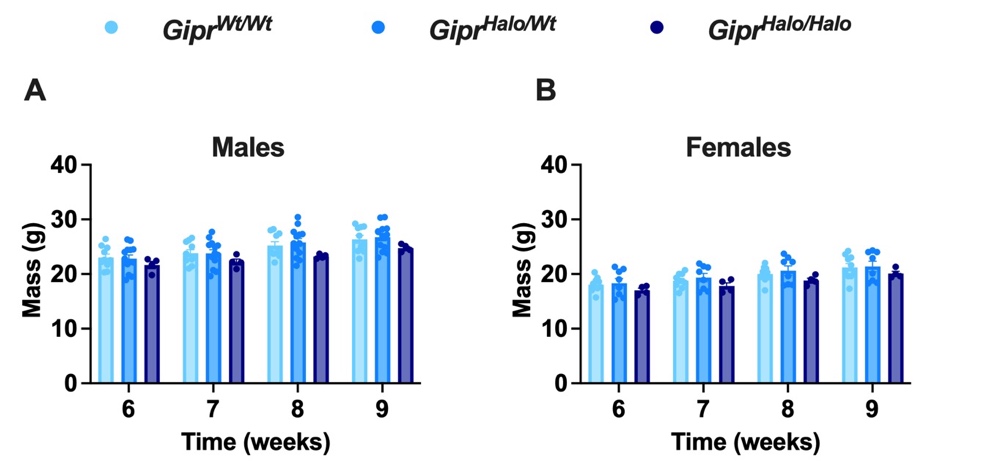


##### **Supplementary Figure 1: No difference in body weights between *Gipr^Wt/Wt^*, *Gipr^Halo/Wt^* and *Gipr^Halo/Halo^* mice at 6-9 weeks of age.**

**A**) Body mass (g) of *Gipr^Wt/Wt^* (male: n=10, female: n=10), *Gipr^Halo/Wt^* (male: n=13, female: n=8) and *Gipr^Halo/Halo^* (male: n=4, female: n=4), measured at 6-9 weeks of age. Males are displayed in **A** and females in **B.** Data was analysed using a two-way ANOVA with time and genotype as co-variables. Šídák test used to correct for multiple comparisons. Values are presented as a mean ± SEM.


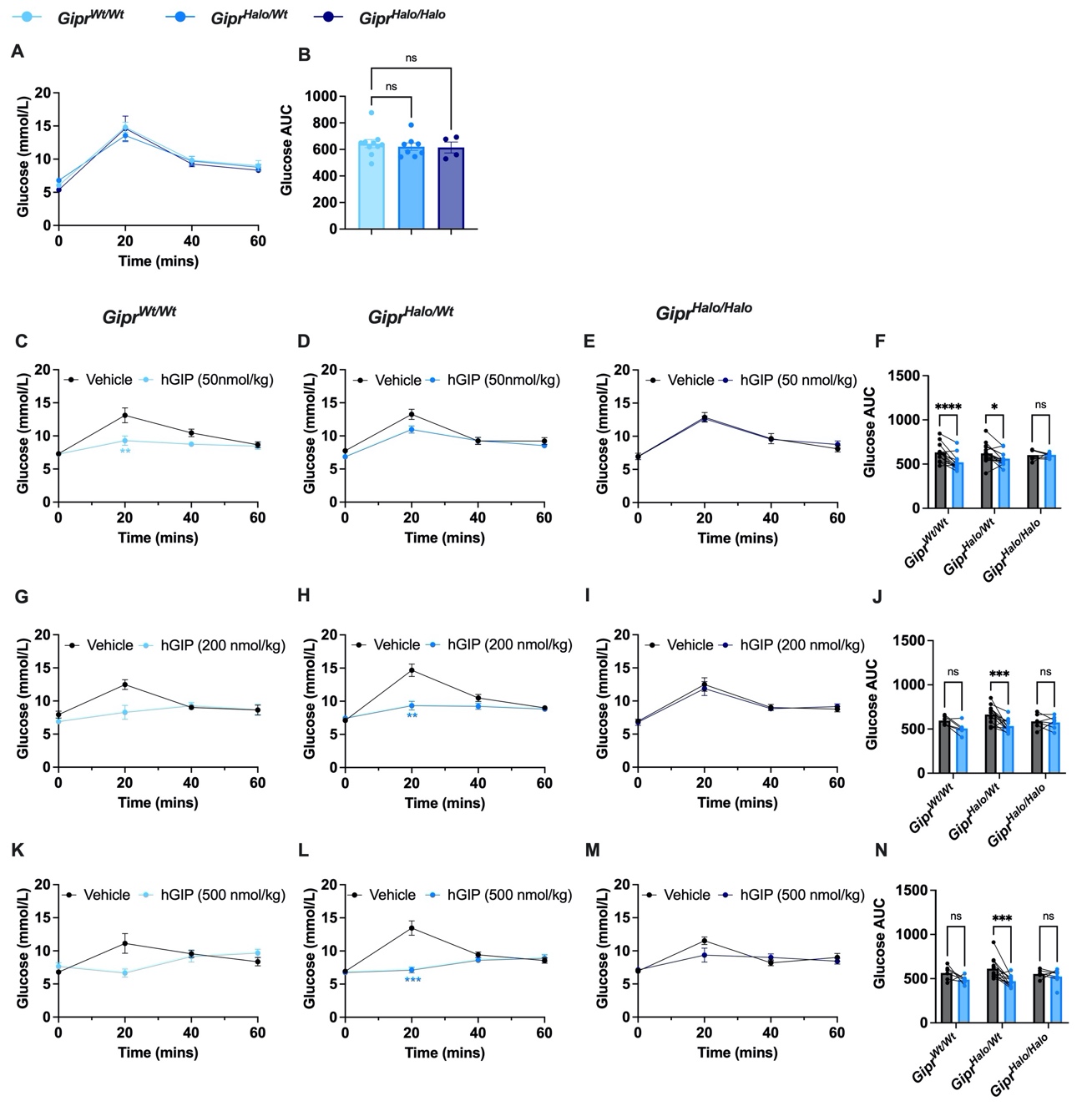


**Supplementary Figure 2: N-terminally Halo tagged GIPR displays a partially impaired receptor function *in vivo* in female mice.**

**A**, **B**) OGTT conducted in *Gipr^Wt/Wt^* (n=10), *Gipr^Halo/Wt^* (n=8) and *Gipr^Halo/Halo^* (n=4) female mice. **C**-**N**) Crossover IPGTTs conducted in *Gipr^Wt/Wt^* female mice (**C**: n=13, **G**: n=6, **K**: n=6), *Gipr^Halo/Wt^* female mice (**D**: n=16, **H**: n=13, **L**: n=13) and *Gipr^Halo/Halo^* female mice (**E**: n=7, **I**: n=7, **M**: n=6-7), in response to human GIP (hGIP) 50 nmol/kg (**C**-**F**), hGIP 200 nmol/kg (**G-J**) and hGIP 500 nmol/kg (**K**-**N**). **A**, **C**-**E**, **G**-**I**, **K**-**M**) Plasma glucose time-course. **B**, **F**, **J**, **N**) Glucose AUC derived from corresponding glucose curves. Blood glucose at specific time-points have been analysed using a two-way ANOVA with time and subgroup as co-variables. Šídák test was used to correct for multiple comparisons. Glucose AUC in **B** has been analysed with a one-way ANOVA. Dunnett’s test was used to correct for multiple comparisons. Glucose AUCs in **F, J** and **N** have been analysed using a two-way ANOVA with genotype and subgroup as co-variables. Šídák test was used to correct for multiple comparisons. Values are presented as a mean ± SEM. *P<0.05, **P<0.01, ***P< 0.001, ****P< 0.0001.


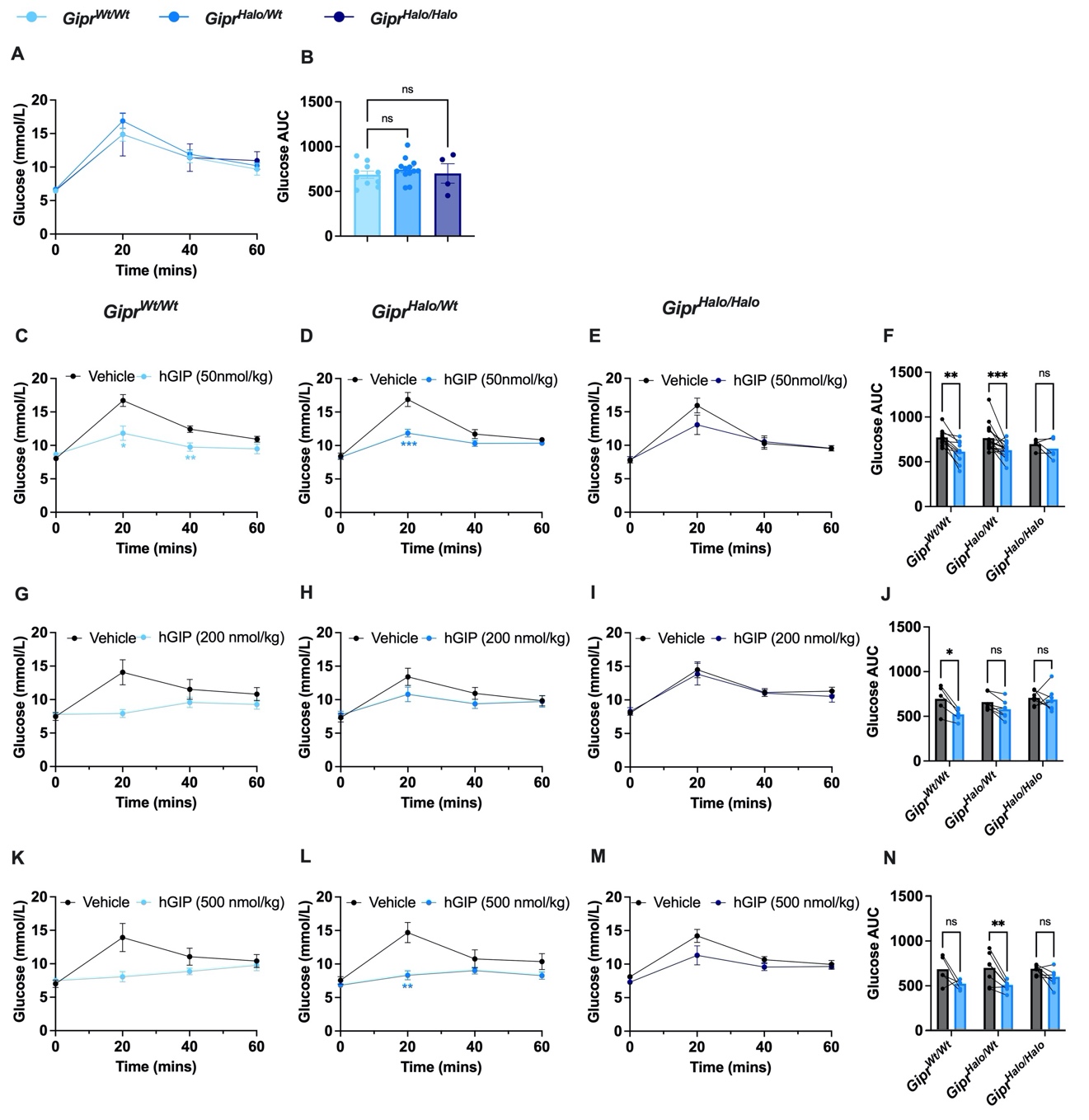


**Supplementary Figure 3: N-terminally Halo tagged GIPR displays a partially impaired receptor function *in vivo* in male mice.**

**A**-**B**) OGTT conducted in *Gipr^Wt/Wt^* (n=10), *Gipr^Halo/Wt^* (n=13) and *Gipr^Halo/Halo^* (n=4) male mice. **C**-**N**) Crossover IPGTTs conducted in *Gipr^Wt/Wt^* male mice (**C**: n=11, **G**: n=5, **K**: n=5), *Gipr^Halo/Wt^* male mice (**D**: n=17, **H**: n=7, **L**: n=7) and *Gipr^Halo/Halo^* male mice (**E**: n=5, **I**: n=7, **M**: n=7), in response to human GIP (hGIP) 50 nmol/kg (**C**-**F**), hGIP 200 nmol/kg (**G**-**J**) and hGIP 500 nmol/kg (**K**-**N**). **A**, **C**-**E**, **G**-**I**, **K**-**M**) Plasma glucose time-course. **B**, **F**, **J**, **N**) Glucose AUC derived from corresponding glucose curves. Blood glucose at specific time-points have been analysed using a two-way ANOVA with time and subgroup as co-variables. Šídák test was used to correct for multiple comparisons. Glucose AUC in **B** has been analysed with a one-way ANOVA. Dunnett’s test was used to correct for multiple comparisons. Glucose AUCs in **F, J** and **N** have been analysed using a two-way ANOVA with genotype and subgroup as co-variables. Šídák test was used to correct for multiple comparisons. Values are presented as a mean ± SEM. *P<0.05, **P<0.01, ***P< 0.001.


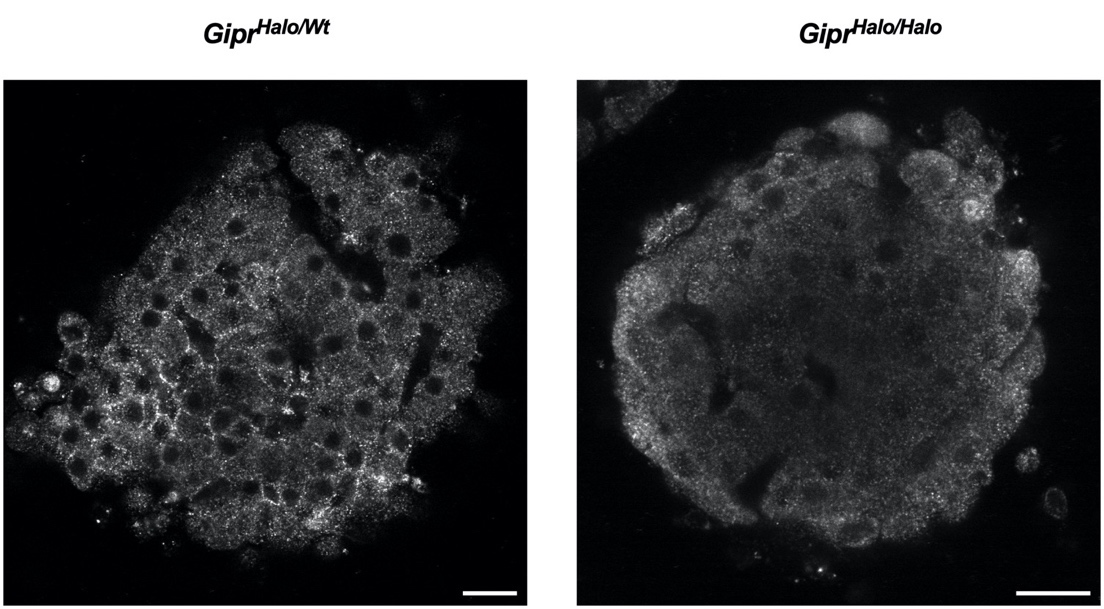


**Supplementary Figure 4: Anti-Halo staining in *Gipr^Halo/Halo^ versus Gipr^Halo/Wt^* mouse islets.** Representative images of a *Gipr^Halo/Wt^* mouse pancreatic islet compared to a *Gipr^Halo/Halo^* mouse pancreatic islet labelled with an anti-Halo antibody. Scale bars = 20 μm.
